# Lennox-Gastaut and Schizophrenia: Comorbidity or complication?

**DOI:** 10.1192/j.eurpsy.2022.1974

**Published:** 2022-09-01

**Authors:** Z. Bencharfa, Y. Amara, L. Tbatou, S. Belbachir, A. Ouanass

**Affiliations:** University Hospital Center Ibn Sina, Ar-razi Psychiatric Hospital, Salé, Morocco

**Keywords:** Psychosis, Lennox-gastaut, evolution

## Abstract

**Introduction:**

Lennox-Gastaut syndrome (LGS) belongs to the group of severe childhood epileptic encephalopathies and represents 1 to 2% of all childhood epilepsies.It is characterized by the occurrence of generalized epileptic seizures, characterized by a particular pattern of the electroencephalogram; slowed mental development and personality disorders.

This syndrome appears between the ages of 2 and 7 years, and its management remains difficult, as it is generally refractory to conventional treatment.The long-term prognosis of this syndrome is poor, marked by the presence of periods of regression of cognitive functions, the appearance of frontal or even psychotic signs and extrapyramidal and cerebellar signs.

**Objectives:**

We will try trow a clinical case, to discuss the evolution of Lennox Gastaut syndrome towards schizophrenia, which remains an infrequent complication, and to determine what would be the adequate management of these patients?

**Methods:**

We report the case of a 16-year-old patient,followed for Lennox Gastaut syndrome since the age of 03, who presented to the psychiatric emergency room for psychomotor agitation, geophagia and altered general condition. The admission interview showed a patient with motor instability, disorganized speech, delusional persecution syndrome, auditory and intrapsychic hallucinations, suicidal ideations in the context of mental automatism, impaired judgment and insight, and insomnia.

The blood tests and the brain CT scan came back without any particularities.

**Results:**

The patient was put on Risperidone, Valproate sodium, Lamotrigine and Clobazam, with good clinical evolution.

**Conclusions:**

The cognitive consequences are catastrophic, 85 to 92% of the patients have a progressive cognitive deterioration, in spite of the reduction of the frequency of the seizures and the improvement of the paroxysmal EEG anomalies.

**Disclosure:**

No significant relationships.

